# Long-term stability of ocular function after alloplastic computer-aided design/computer-aided manufacturing reconstruction of posttraumatic orbital defects: a retrospective cohort study

**DOI:** 10.1186/s13005-025-00582-x

**Published:** 2026-01-28

**Authors:** S Skade, ML Linderkamp, F Lentge, P Korn, P Jehn, NC Gellrich, K Hufendiek, R Zimmerer, MT Neuhaus

**Affiliations:** 1 Department of Oral and Maxillofacial Surgery, Hannover Medical School, 30625 Hannover, Germany; 2 Department of Ophthalmology, Hannover Medical School, 30625 Hannover, Germany; 3 Department of Oral and Maxillofacial Surgery, University Hospital Tuebingen, 72076 Tuebingen, Germany

**Keywords:** Computer-aided design/Computer-aided manufacturing, Orbital floor reconstruction, Eyeball position, Visual acuity, Ocular motility, Diplopia, Titanium implant

## Abstract

**Background:**

We investigated the long-term stability of ocular function and eyeball position after posttraumatic orbital reconstruction of patient-specific implants using alloplastic computer-aided design/computer-aided manufacturing (CAD/CAM). In this study, we evaluated the extent to which precise anatomical reconstruction contributes to stable functional and aesthetic outcomes over time.

**Methods:**

Between 2010 and 2018, we retrospectively analyzed 69 patients who underwent orbital floor and medial wall reconstruction. The patients were classified into two groups following the implant type: CAD/CAM-manufactured titanium implants and standard CAD-based titanium implants. Long-term follow-up (LTFU) assessments included globe positioning, diplopia, motility, visual acuity, and intraocular pressure. The differences between the two groups were determined using statistical analyses.

**Results:**

Deviations in the sagittal and vertical globe positions persisted after a median follow-up of 56 months in both groups, with no significant differences. Vertical globe deviation is significantly associated with diplopia. Patients with CAD-based implants showed a higher tendency for motility restriction and lower visual acuity than those with CAD/CAM implants. Intraocular pressure was significantly lower in the affected eyes in both groups.

**Conclusion:**

CAD/CAM-designed implants provide improved anatomical reconstruction; however, they do not significantly improve long-term globe positioning than CAD-based implants. However, they may also contribute to improved motility and visual outcomes. A close interdisciplinary approach involving ophthalmologists is important for optimizing treatment planning and postoperative assessments.

## Introduction

Enophthalmos and hypoglobus are among the most common clinical symptoms of posttraumatic orbital deformities [1–3]. Restoring the shape of the infraorbital rim and orbital floor when reconstructing the orbit is important, to avoid the typical dystopia of the globe. Before patient-specific biomodels became available, implants could be pre- or intraoperatively bent on a standard skull. Currently, there are two different types of titanium implants, both based on a three-dimensional CT or CBCT data set. One established technique is the use of standardized titanium mesh implants, which are manually pre-bent preoperatively on a patient-specific, 3D-printed biomodel. In contrast, there are completely patient-specific CAD/CAM-manufactured implants, which are modeled by mirroring the healthy orbit using the CT or CBCT scan [4]. Reportedly, orbit reconstruction using computer-aided design/computer-aided manufacturing (CAD/CAM) patient-specific implants and intraoperative navigation has increased the accuracy of orbital volume reconstruction and avoided these severe complications [4–6]. Nevertheless, orbital implants prebent on an in-house printed biomodel can remain an adequately fast and cost-sensitive solution [7]. Other materials used in orbital surgery, such as PEEK, PDS, or bone grafts, will not be discussed here.

Further enhancements in CAD/CAM implant design, as shown in other fields of maxillofacial surgery [8, 9], have been used in the design of orbital implants. By adding stabilizing extensions in the regions of anatomical landmarks to the design of orbital implants, the design itself forms a “one-fit-only” position. This will prevent initial malpositioning of the implant, and also permit better visual and haptic feedback for the surgeon [6]. These sophisticated CAD/CAM orbital implants can be considered the gold standard for alloplastic orbital reconstruction together with intraoperative navigation and intraoperative cone-beam computed tomography (CBCT) [5, 7]. Introducing intraoperative CBCT can further reduce the risk of revision surgery [10, 11].

However, medical evidence on the long-term course and stability of postoperative outcomes, particularly regarding the use of patient-specific implants are limited.

In this study, we aimed to obtain data on the recovery and stability of ocular function and eyeball position after reconstruction of posttraumatic defects of the orbital floor and medial wall. Following these results, assessing the extent to which faithful reconstruction contributes to long-term stability and to what extent initial overcorrections of the original volume are indicated should be possible.

## Materials and methods

### Ethical background

This study was approved by the local ethics review committee (Hannover Medical School; study no.: 8163_BO_K_2018). The analyses were performed in accordance with the declaration of Helsinki. All included patients provided informed consent.

### Study protocol

The Department of Oral and Maxillofacial Surgery at the Hannover Medical School database was screened for patients treated for orbital floor or wall fractures between 2010 and 2018.

### Inclusion criteria were:

- Legal age
- Surgical reconstruction of orbital floor or wall fractures
- Pre- and postoperative 3D data (computed tomography (CT) or CBCT)
- The follow-up interval was at least 4 months.

Most patients who underwent posttraumatic orbital reconstruction at the Department of Oral and Maxillofacial Surgery of Hannover Medical School (MHH) underwent annual checkups regularly after the initial, more frequent follow-up visits. Preoperative diagnostics routinely include 3D imaging using CT or CBCT.

Sixty-nine patients met the inclusion criteria. Fifty-two of the included patients were already part of the multicenter “Orbita3”-study, conducted between 2010 and 2014. The AOCMF (AO Foundation, Davos, Switzerland) funded this study, and the study was sponsored by the AO Clinical Investigation and Documentation (AOCID). This study was registered in Clinical Trials. gov under the code NCT01121159.

The patients included in this study were divided into two cohorts based on the type of implant used for reconstruction. Patients whose orbital defect was reconstructed using a patient-specific CAD/CAM-manufactured titanium implant (KLS Martin, Tuttlingen, Germany) (hereafter referred to as “CAD/CAM-based individualized”) were in group one. Patients who were reconstructed using a standard orbital floor implant (Synthes^®^ CMF, West Chester, PA, USA) attached to an individual patient model (Phacon GmbH, Leipzig, Germany) (hereafter referred to as “CAD-based individualized”) were in group two. They received orbital floor mesh plates with a thickness of 0.2 mm, 0.3 mm, or 0.4 mm. With the exception of three patients in the CAD/CAM group, all patients underwent primary reconstruction.

Preoperative intervention planning was conducted in both cohorts using iPlan CMF (version 3.0.5; BrainLab, Feldkirchen, Germany).

### Statistical analysis

The demographic data and surgical details were summarized using standard descriptive statistics (mean and standard deviation or median and interquartile range for continuous variables and absolute number and frequency distribution for categorical variables). The Mann–Whitney U test, Wilcoxon Singed-Rank test, and Chi-Square test were used to determine the two group’s statistically significant differences. Statistical significance was set at *p* <.05. Microsoft Excel 2019 (Microsoft^®^, Washington DC, USA) was used to prepare and manage the data. All statistical analyses were conducted using SPSS version 26 (IBM Corp., Armonk, NY, USA).

## Results

### Demographics

The mean age of the 69 patients was 44 years at the time of surgery with a standard deviation of 17 years. The male-to-female ratio was about 2:1. Orbital fractures were majorly caused by traffic accidents in 22 patients (31.4%). The two groups were not significantly different in the circumstances of the accident. However, significant differences were found when comparing men and women. The right and left orbits were affected in 49 and 30 patients, respectively.

**Fig. 1:**
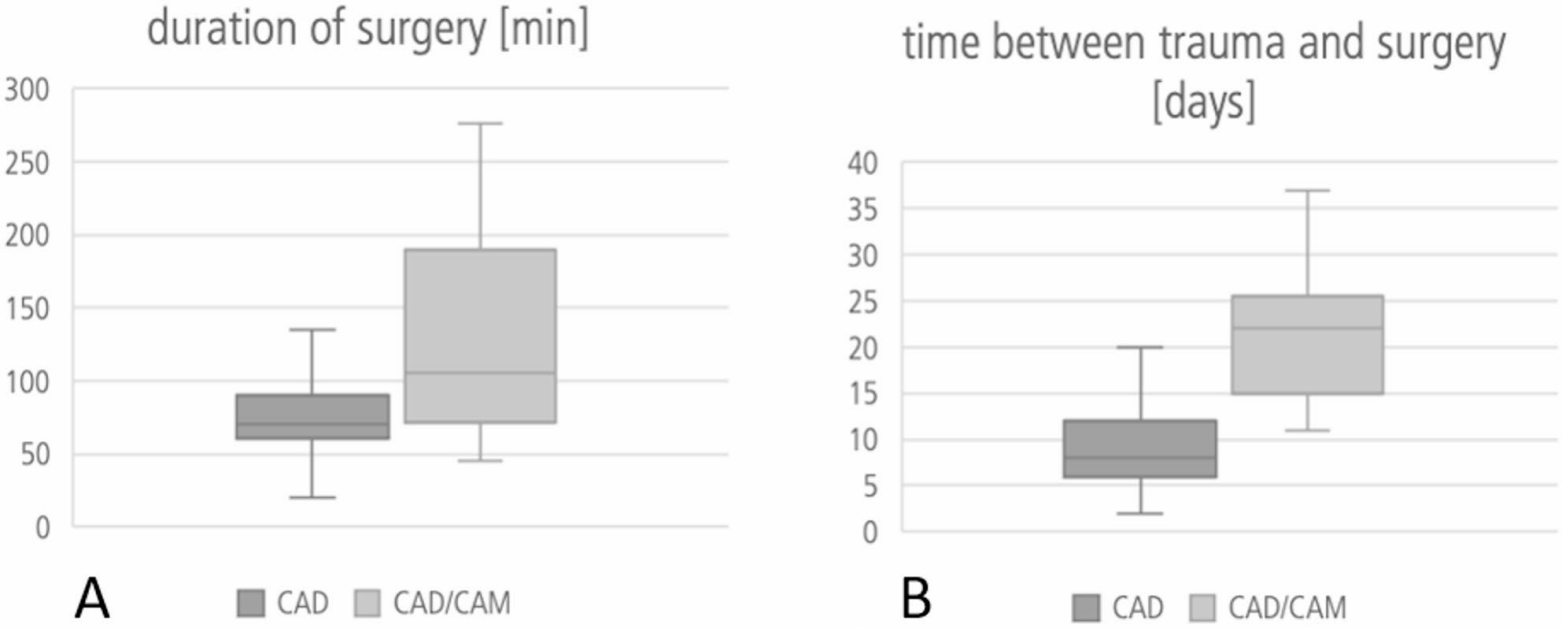
Time of surgery and Time between trauma and surgery (**A**) Boxplot of surgery duration for both treatment groups (median and standard deviation), **B** Boxplot of days between initial trauma and date of surgery for both treatment groups (median and standard deviation)

Most patients underwent surgery within the first 2 weeks of the accident (*n* = 44). The time difference between the day of the accident and surgery was significantly (*p* <.01 Mann-Whitney U test) different between both groups (CAD vs. CAD/CAM) (Fig. 1B). While 87.5% of the CAD group underwent surgery within two weeks of the accident, most of the CAD/CAM group underwent surgery later. About 61.9% of patients in the CAD/CAM group underwent surgery within a month. The duration of the operation (incision-suture time) was significantly shorter in patients who received a CAD implant (*p* =.017 Mann-Whitney U test) (Fig. 1A).

### Clinics

Follow-up examinations were conducted after 1–8 years of the orbital floor’s surgical reconstruction. The timing of long-term follow-up (LTFU) varied among the patients. The median LTFU period was 56 months (mean, 53 months). We lost some of the patients to follow-up.

### Vertical and sagittal globe position

The difference in globe position was measured in the sagittal and vertical directions. The affected eye was compared with the unaffected eye. In the case of sagittal bulbar position, we refer to anteroposterior alignment. The difference was measured using a Naugle enophthalmometer. To measure the differences in vertical eyeball position between the affected and unaffected eyes, we first determined whether the bipupillary line descended to one side. A reference line was determined at a right angle to the center of the face, and then the offset to the center of the pupil was measured. The vertical direction refers to the craniocaudal direction. In 33 of the 42 patients, we found a deviation in the sagittal globe position compared with the unaffected side at LTFU. We also found a change in the vertical globe position in 25 patients (Table 1). However, both groups did not significantly differ. The average difference in the sagittal globe position between the affected and unaffected eye was 0.3 mm in the CAD group and 1.0 mm in the CAD/CAM group.

**Table 1:** Occurrence of Globe dystopia between affected and unaffected eye

Change of vertical globe position LTFU affected > unaffected	CAD-based individualized	CAD/CAM-based individualized	*p* Value
n (%)	22	20	.231^a^
No	7 (31.8)	10 (50.0)	
Yes	15 (68.2)	10 (50.0)	

Change of sagittal globe position LTFU affected > unaffected			
n (%)	22	20	.591^a^
No	4 (18.2)	5 (25.0)	
Yes	18 (81.8)	15 (75.0)	
^a^Chi-Square test			

*CAD* Computer-aided design, *CAM* Computer-aided manufacturing, *LTFU* long-term follow-up

### Double vision

At LTFU, 13 patients (31.7%) had double vision (Table 2). Eight of these patients were from the CAD group, and five were from the CAD/CAM group. The occurrence of double vision based on patient age was not significantly different. Half of the patients who showed a change in the vertical position of the globe reported double vision (*p* =.003 Chi-Square test). Only 28.1% of patients with a change in the sagittal globe had double vision (*p* =.353 Chi-Square test). None of the patients underwent surgery on the external eye muscles due to double vision.

**Table 2:** Occurrence of diplopia during LTFU

Diplopia LTFU	CAD-based individualized	CAD/CAM-based individualized	*p* Value
n (%)	22	19	.491^a^
No	14 (63.6)	14 (73.7)	
Yes	8 (36.4)	5 (26.3)	
^a^ Chi-Square test			

*CAD* Computer-aided design, *CAM* Computer-aided manufacturing

### Motility

Ocular motility was measured using Kestenbaum limbus test. Four lines of sight were observed (upgaze, downgaze, abduction and adduction) and the affected eye was compared with the unaffected eye. Restrictions were classified into four qualities (none, slight, moderate, and severe). No difference was rated the as “None”, a difference of 1–2 mm was rated as “slight”. A difference between 3 and 4 mm was rated as “moderate” and 5 mm or more as “severe”. This examination was dependent on the patient’s cooperation. Not all the measured values were usable, explaining the aberrant number of cases (Table 3). No significant differences were observed between the groups. Most patients showed no restrictions in ocular motility.

**Table 3:** Occurrence of ocular motility disturbance during LTFU

Direction of movement and level of impairment		CAD-based individualized (*n* = 18)	CAD/CAM-based individualized (*n* = 16)		*p* Value
Upgaze	none	11 (61.1)	12 (75.0)		0.388 ^a^
	slight	7 (38.9)	4 (25.0)		
	moderate	0	0		
	severe	0	0		
Downgaze	none	10 (55.5)	9 (56.3)		0.626 ^a^
	slight	7 (38.9)	7 (43.7)		
	moderate	0	0		
	severe	1 (5.5)	0		
Abduction	none	12 (66.7)	15 (93.8)		0.143 ^a^
	slight	5 (27.8)	1 (6.2)		
	moderate	1 (5.5)		0	
	severe	0		0	
Adduction	none	14 (77.8)		15 (93.8)	0.189 ^a^
	slight	4 (22.2)		1 (6.2)	
	moderate	0		0	
	severe	0		0	
*Chi-Square test					

*CAD* Computer-aided design, *CAM* Computer-aided manufacturing

### Visual acuity

Near and distant visual acuities were examined to compare the visual acuity between the affected and unaffected eyes (Table 4). The examination was performed without visual acuity correction. The distant visual acuity of the affected eye was significantly reduced more often in the CAD group than in the CAD/CAM group.

**Table 4:** Near and distant visual acuity during LTFU

Near Affected < unaffected			
	CAD-based individualized	CAD/CAM-based individualized	*p* Value
n (%)	22	19	.138^a^
No	14 (63.6)	16 (84.2)	
Yes	8 (36.4)	3 (15.8)	

Distant Affected < unaffected			
	CAD-based individualized	CAD/CAM-based individualized	*p* Value
n (%)	20	19	.023^a^
No	10 (50.0)	16 (84.2)	
Yes	10 (50.0)	3 (15.8)	
^a^ Chi-Square test			

CAD Computer-aided design, *CAM* Computer-aided manufacturing

### Intraocular pressure

Intraocular pressure was estimated using air puff tonometry. The values for the affected eyes were compared with those for the unaffected eyes (Table 5). The CAD and CAD/CAM groups were not significantly different. The intraocular pressure of the affected eye in both groups was lower than that of the unaffected eye (*p* =.858 Chi-Square test). If the measured pressures were considered independent of the implant type, there would be a significant difference between the affected and unaffected eyes. The pressure in the affected eye was significantly reduced (*p* =.038 Wilcoxon Signed-Rank test).

**Table 5:** Intraocular pressure between affected and unaffected eye in LTFU

	CAD-based individualized	CAD/CAM-based individualized	*p* Value
n (%)	19	18	.858^a^
Affected = non-affected	2 (10.5)	1 (5.6)	
Affected < non-affected	11 (57.9)	11 (61.1)	.038^b^
Affected > non-affected	6 (31.6)	6 (33.3)	
^a^ Chi-Square test ^b^ Wilcoxon Signed-Rank test			

*CAD* Computer-aided design, *CAM* Computer-aided manufacturing

## Discussion

In this study, we aimed to assess the possible difference in postoperative outcomes, especially at LTFU, between orbital implants of different individualization grades. A limitation of this study is the small sample size, which may reduce the statistical power and generalizability of the results. Some data were missing because we lost some patients to follow-up, which could have introduced bias into the analysis. The findings may not be transferable to other cohorts or settings, and potential confounding variables were not fully controlled. The results of this study show that aberrations in the globe position persisted in both groups even during LTFU, independent of the orbital implant type. Patients with CAD implants showed deviations of the affected eye in the vertical and sagittal positions more often than patients with CAD/CAM implants. However, statistically, the difference was insignificant. In this study, a change in the vertical position of the eyeball was significantly more often related to the occurrence of double vision than changes in the sagittal position. The vertical globe position seems to be more relevant to the occurrence of double vision and is thus more important during orbital reconstruction. The occurrence of vertical globe dystopia was slightly higher in the CAD group than in the CAD/CAM group, further indicating that anatomically correct reconstruction of the orbital floor is eased by more accurate preoperative three-dimensional planning and a higher grade of individualized orbital implant design, as proven by many authors [5, 12–14]. In a former work of our group, we were able to show that the implant position does not correlate with patient complaints [12]. A higher grade of patient specificity in orbital floor reconstruction with CAD/CAM-based implants did not result in significantly better positioning of the eyeball in our study; however, the gentler insertion of the functionalized CAD/CAM implants can possibly affect the condition of the soft tissue also [5, 12]. In this regard, this study also showed a likelihood in the motility of the globe that favors CAD/CAM-based implants. Patients with CAD-based implants tend to show motility restrictions more frequently. In their study, Zimmerer et al. showed that patients who received a patient-specific individualized implant had lesser double vision and motility impairments. We showed that this was also the case at the time of LTFU and that the results remained stable over time. This might be linked to less traumatic implant insertion in the CAD/CAM-based group and less scarring of the periorbital tissue, resulting in better ocular motility.

Contrary to expectations, the duration of surgery (time between incision and suture) was significantly shorter in patients who received a CAD implant. Due to the high degree of customization of CAD/CAM implants, the duration of surgery should decrease significantly. We attribute this observation to the fact that the cases examined here fall within the early stages of what was then a new surgical technique (fully individualized CAD/CAM patient-specific implants).

Half of the patients with CAD-based implants showed poorer distance vision in the affected eye than in the unaffected eye. Kim et al. analyzed the frequency of reduced visual acuity in patients with blowout and non-blowout fractures [15]. They reported frequencies of 7.5% and 8.3%, respectively, with no distinction made between the affected and unaffected eyes. Studies have concentrated on the occurrence of optic nerve neuropathy and blindness after orbital trauma [16–18]. The fact that the visual acuity of the affected eye in the CAD group was often worse than that of the healthy eye is an interesting observation that should be verified using more detailed preoperative examinations.

Midfacial fracture management aims to achieve prompt reconstruction [19, 20]. However, CAD/CAM-individualized implant production still takes time. Therefore, it takes about 2–4 weeks to receive treatment in the Department of Oral and Maxillofacial Surgery at Hannover Medical School. Reconstruction was conducted after an average of eight and 33 days in the CAD and CAD/CAM groups, respectively. In a meta-analysis by Jazayeri et al. in 2020, patients who underwent surgery within 2 weeks after trauma were more likely to fully recover from all symptoms and less likely to experience postoperative double vision and enophthalmos [21]. However, we were unable to conduct this observation in this study. Some authors recommend a wait-and-see approach in cases of milder clinical symptoms of posttraumatic orbital defects to reevaluate the evidence for a surgical procedure once the edema and hematoma have been reduced [22, 23]. The Orbital Assessment Algorithm (OA^2^) was confirmed at the Department of Orbital and Maxillofacial Surgery at the MHH to ease the decision-making process for or against the surgical treatment of an orbital floor defect and ensure treatment quality [7]. Understanding that it is about treating the defect, not the fracture, the treatment decision depends on the patient’s clinical pathology and radiographic results. If no emergency intervention is needed, a patient-specific implant can be scheduled without the time required, which is disadvantageous for the patient, as shown in this study. In cases of late reductions that are necessary, the fixation of the bony outer frame is reduced and internally fixed in the first step. Once the swelling and hematoma were reduced, the indication for surgery was re-evaluated approximately 2 weeks after the primary surgery. Subsequently, early secondary orbital reconstruction is conducted based on the patient’s clinical symptoms (dystopia of the globe and diplopia).

## Conclusion

In the next step towards CAD/CAM, further enhancements in the quality management and reproducibility of preoperative planning are expected. The achievements of 3D imaging and its advantages in orbital surgery are evident; however, they are yet to reach all operating theatres. A disagreement regarding the evidence-based need for CAD/CAM orbital reconstruction remains. In this context, there is a need for parameters that permit the reliable prediction of patient outcomes with or without surgical treatment. Until then, CAD-based implants remain a quick and affordable alternative in cases where rapid intervention is required or the necessary technical capabilities are lacking. It is important to us that this type of injury should be treated in close collaboration with specialties from ophthalmology. An ophthalmological examination that goes beyond the usual methods used in oral and maxillofacial surgeries seems reasonable. Further studies should focus on routine consultation with ophthalmologists.

## Data Availability

The datasets generated and analyzed during the current study are not publicly available due to institutional restrictions but are available from the corresponding author upon reasonable request.

## Abbreviations

CAD: computer-aided design
CAM: computer-aided manufacturing
LTFU: long-term follow up
CBCT: cone-beam computed tomography
CT: computed tomography
AOCID: AO Clinical Investigation and Documentation
OA^2^: Orbital Assessment Algorithm
3D: three dimension/three-dimensional
